# Self-assembled growth of MnSi_~1.7_ nanowires with a single orientation and a large aspect ratio on Si(110) surfaces

**DOI:** 10.1186/1556-276X-8-45

**Published:** 2013-01-22

**Authors:** Zhi-Qiang Zou, Wei-Cong Li, Xiao-Yong Liu, Gao-Ming Shi

**Affiliations:** 1 Centre for Analysis and Testing, Shanghai Jiao Tong University, 800 Dongchuan Road, Shanghai, 200240, China; 2 Department of Physics, Shanghai Jiao Tong University, 800 Dongchuan Road, Shanghai, 200240, China

**Keywords:** Self-assembled growth, Nanowires, Transition metal silicides, Scanning tunneling spectroscopy, Silicon (110).

## Abstract

MnSi_~1.7_ nanowires (NWs) with a single orientation and a large aspect ratio have been formed on a Si(110) surface with the molecular beam epitaxy method by a delicate control of growth parameters, such as temperature, deposition rate, and deposition time. Scanning tunneling microscopy (STM) was employed to study the influence of these parameters on the growth of NWs. The supply of free Si atoms per unit time during the silicide reaction plays a critical role in the growth kinetics of the NWs. High growth temperature and low deposition rate are favorable for the formation of NWs with a large aspect ratio. The orientation relationship between the NWs and the reconstruction rows of the Si(110) surface suggests that the NWs grow along the $\left[1\bar{1}0\right]$ direction of the silicon substrate. High-resolution STM and backscattered electron scanning electron microscopy images indicate that the NWs are composed of MnSi_~1.7_.

## Background

Self-assembled nanowires (NWs) of metal silicides have received much attention recently for their potential applications as electrical interconnects on a scale that cannot be attained with conventional lithographic methods [1-4]. In addition, such structures are expected to display novel physical properties related to the structural anisotropy and quantum confinement effects and could be used as active elements for the new generation of electronic, optoelectronic, magnetic, and thermoelectric devices [5-7]. In the past decade, it has been reported that NWs of rare-earth silicides such as ScSi_2_[7], ErSi_2_[8,9], DySi_2_[2,10,11], GdSi_2_[12,13], and HoSi_2_[14,15] and 3*d* transition metal silicides such as FeSi_2_[1], CoSi_2_[3], NiSi_2_[16], and TiSi_2_[17-19] can be formed on silicon substrates by the molecular beam epitaxy method. While the NW shape of rare-earth silicides is thought to result from an anisotropic lattice mismatch that is small (<1%) in length direction and large (>5%) in width direction of the NW, the NW shape of FeSi_2_, CoSi_2_, and NiSi_2_ results from an ‘endotaxial’ growth mechanism which involves the growth of silicide into the Si substrate [1,3].

Very recently, we have reported that MnSi_~1.7_ NWs can also be grown on the Si substrates with reactive epitaxy method at temperatures above approximately 500°C [20-22]. The growth mechanism of the NWs was considered to be anisotropic lattice mismatch between the silicide and the Si substrates. The growth direction of the NWs is confined along Si<110>, resulting in the NWs orienting with the long axis along one direction (Si$\left[1\bar{1}0\right]$), two orthogonal directions (Si$\left[01\bar{1}\right]$ and [011]), and three directions (Si$\left[01\bar{1}\right]$, $\left[01\bar{1}\right]$, and $\left[1\bar{1}0\right]$) on the Si(110), (001), and (111) surfaces, respectively. However, for scientific investigation as well as device applications, it would be highly expected to grow NWs with a single orientation because the NWs grown in this mode would never cross and have larger length. Parallel NW arrays can be used as nanomechanical devices [23], and using parallel NWs, the anisotropic electronic structure of silicide NWs can be investigated by angle-resolved photoelectron spectroscopy [11]. On the other hand, the Si(110) surface is currently attracting renewed interests because of its unusual properties such as high hole mobility, unique surface reactivity, and strong structural anisotropy. The Si(110) surface has a potential use in fabricating vertical double-gate metal oxide semiconductor field effect transistors that enable much higher integration [24]. Although the formation of MnSi_~1.7_ NWs with sole orientation on Si(110) was demonstrated in our previous works [20], a detailed investigation on how the growth parameters affect the growth of MnSi_~1.7_ NWs on Si(110), which is of key importance for a comprehensive understanding of the growth kinetics and thus the controllable growth of the NWs, is still lacking. In this paper, we examine in detail, mainly using scanning tunneling microscopy (STM), the influence of growth temperature, deposition rate, and deposition time on the formation of MnSi_~1.7_ NWs on the Si(110) surface.

## Methods

The experiments were performed in an ultra-high vacuum molecular beam epitaxy-STM system (Multiprobe XP, Omicron, Taunusstein, Germany) with a base pressure of less than 5.0 × 10^−11^ mbar. Substrates used for the deposition were cut from a phosphorus-doped, n-type Si(110) wafer with resistivity of approximately 0.01 Ω cm and have a size of 12 × 2.5 × 0.3 mm^3^. Atomically clean Si(110)-16 × 2 surfaces were prepared by degassing the substrates at about 600°C for 12 h, followed by flashing to 1,200°C and annealing at 600°C for 10 min. Mn was deposited on the Si(110)-16 × 2 surfaces by heating Mn lumps (purity 99.999%) in a Mo crucible with electron bombardment. The Mn flux was monitored by an internal ion collector mounted near the evaporation source. The deposition rate was controlled from approximately 0.01 to 0.5 ML/min (1 ML = 1 metal atom per 1 × 1 surface mesh = 4.78 × 10^14^ Mn atoms/cm^2^) [3]. During deposition, the substrates were heated by radiation from a tungsten filament located at the back of the sample holder. The temperature was set from 450°C to 600°C and measured using a thermocouple. An electrochemically etched tungsten tip was used for scanning. All STM images were recorded at room temperature (RT) with a bias voltage of 2 to 3 V and a tunneling current of 0.1 to 0.2 nA. A backscattered electron scanning electron microscope (BE-SEM) (Nova NanoSEM 230, FEI, Hillsboro, OR, USA) was used to *ex situ* observe the elemental distribution of the samples on a large scale.

## Results and discussion

### Effects of growth parameters on the formation of NWs

**Figure 1:**
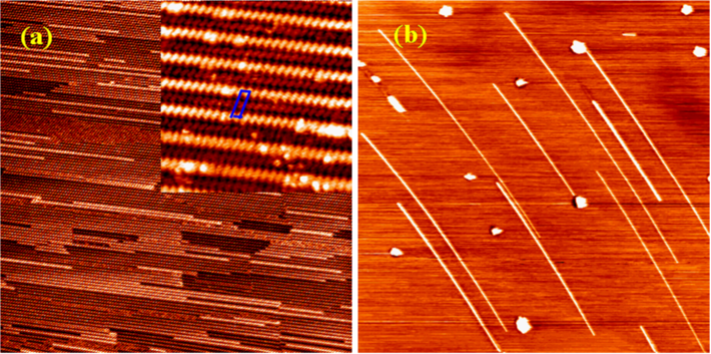
**STM images of the Si(110) surface and the manganese silicide NWs grown on it. (a)** STM images (500 × 500 nm^2^) of a clean Si(110) surface. The inset is a high-resolution STM image (30 × 30 nm^2^) showing the 16 × 2 reconstruction of the surface. A 16 × 2 unit cell is outlined by a rectangle. **(b)** STM image (1,600 × 1,600 nm^2^) of manganese silicide NWs and islands grown by depositing 1 ML Mn on the Si(110) surface at 585°C. During deposition, the deposition rate was kept at approximately 0.02 ML/min.

Figure 1a shows STM images of the atomically clean Si(110) surface obtained by the well-established degassing, flashing, and annealing procedures. The high-resolution image (inset) clearly shows that the surface consists of equally spaced and alternately bright and dark zigzag chains parallel to the $\left[\bar{1}12\right]$ direction, which is the typical characteristic reported for the Si(110)-16 × 2 reconstructed surface [25]. The bright and dark zigzag chains correspond to the upper and lower atomic layers of the Si(110) plane, respectively. The step height between the layers is 1.92 Å. A 16 × 2 unit cell is outlined by a rectangle in the inset.

After surveying the flashed Si(110) surface by STM, we evaporate Mn atoms onto the surface at different substrate temperatures in the range of RT to 600°C, while the deposition rate and time are kept at approximately 0.02 ML/min and 50 min, respectively. We find that only clusters or irregular three-dimensional (3D) islands are formed on the Si(110) surface when the temperature is lower than approximately 475°C. At approximately 475°C, elongated silicide islands begin to form on the surface. With further increasing temperature, the elongated islands grow rapidly in the length direction and remain almost invariant in the width direction, forming a NW-like shape. Meantime, the number density of the NWs is also increased significantly, while that of the 3D islands is decreased. Figure 1b is a typical STM image of the Si(110) surface after deposition at 585°C. It can be seen that straight and parallel NWs with a large aspect (length/width) ratio were formed on the surface. The NWs are about 600 to 1,370-nm long, approximately 18-nm wide, and 2.5-nm high, and their aspect ratios are in the range of approximately 33 to 76.

**Figure 2:**
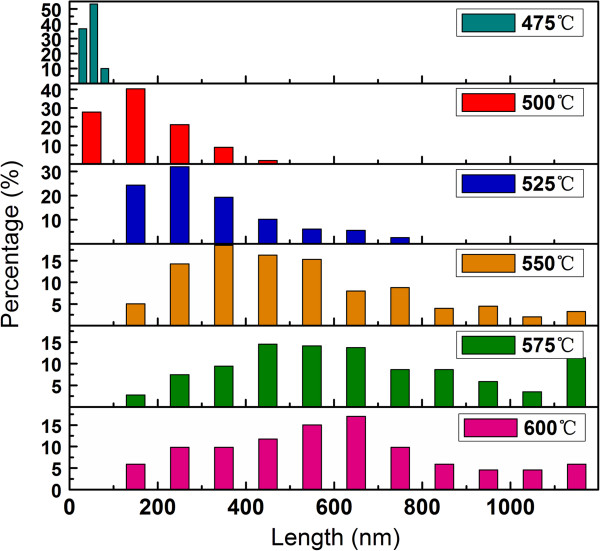
**The length distribution of the manganese silicide NWs formed on the Si(110) surface at different growth temperatures.** During deposition, the Mn deposition rate and coverage were kept at approximately 0.02 ML/min and 1 ML, respectively.

Figure 2 shows the length distribution of the NWs at various growth temperatures. For each temperature, more than 150 NWs were randomly selected from dozens of STM images for statistical purpose. It can be seen that in the range of 475°C to 600°C, the average lengths of the NWs increase with temperature. When the growth temperature is higher than 550°C, 60% and more of the NWs have a length larger than 400 nm, and more than 10% of the NWs have a length exceeding 1.0 μm. In the present work, the aspect ratio of the NWs grown on Si(110) can reach 100, which is larger than that of the NWs formed on a Si(111) surface [21].

**Figure 3:**
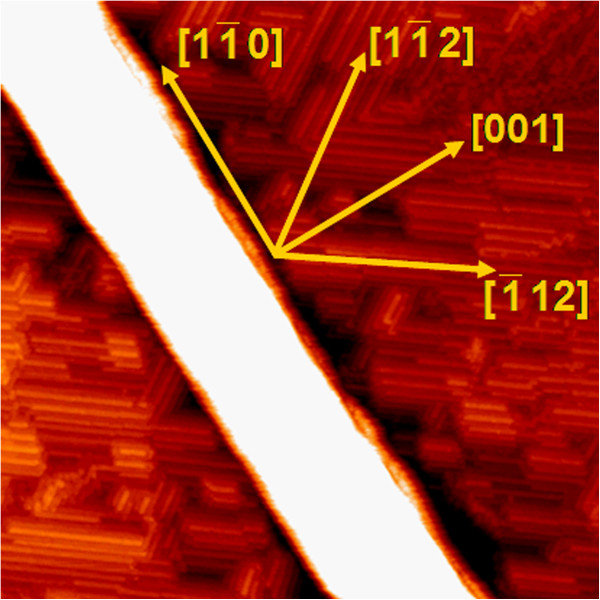
**A typical STM image (200 × 200 nm**^**2**^**) showing the growth direction of the NW.** The reconstruction rows of the Si(110)-16 × 2 surface run along two directions, $\left[1\bar{1}2\right]$ and $\left[\bar{1}12\right]$.

In order to determine the orientation of the NWs on the Si(110) surface, we take a magnified image of a NW, in which the reconstruction rows of the Si(110)-16 × 2 surface can be clearly resolved. The image (Figure 3) shows that the 16 × 2 reconstruction of the Si(110) surface exhibits a double-domain structure with fragmented rows running along two directions, $\left[1\bar{1}2\right]$ and $\left[\bar{1}12\right]$[24], as indicated by the arrows. The angle between the NW edge and the row of the substrate is measured to be 54.7°, which is consistent with the theoretical value of the angle between the $\left[1\bar{1}0\right]$ and the $\left[1\bar{1}2\right]$ directions. Therefore, the NWs are formed on the Si(110) surface with long axis along the $\left[1\bar{1}0\right]$ direction. Similar results were also found in Dy/Si(110) [26] and Fe/Si(110) [1] systems.

**Figure 4:**
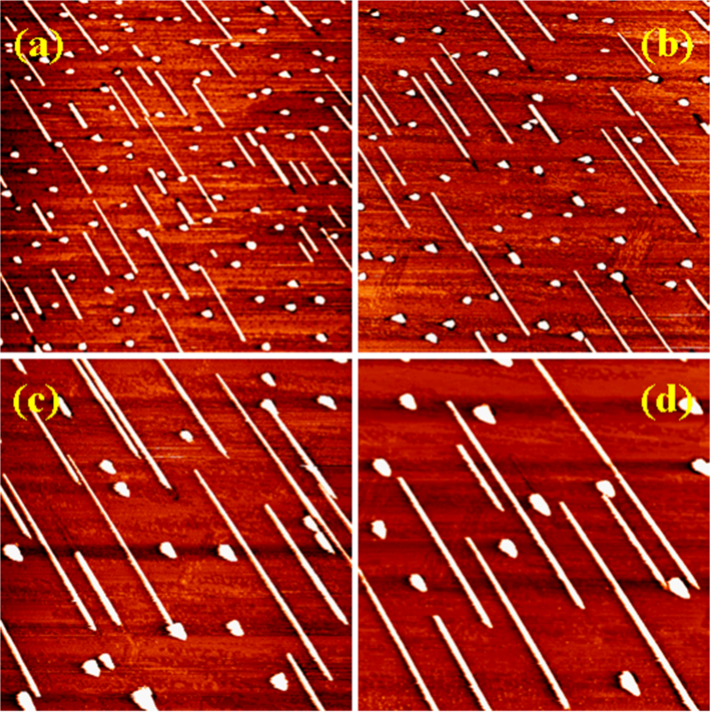
**STM images showing the influence of Mn deposition rate on the growth of NWs.** Series of STM images (1,000 × 1,000 nm^2^) of the manganese silicide NWs and islands grown on the Si(110) surfaces at various depositing rates. (**a**) Approximately 0.02, (**b**) 0.05, (**c**) 0.2, and (**d**) 0.5 ML/min. The growth temperature and the Mn coverage were kept at 550°C and 1 ML, respectively.

Figure 4 is a series of STM images showing the influence of Mn deposition rate on the growth of NWs, with the temperature and Mn coverage kept at 550°C and 1 ML, respectively. The statistical results of the dimensions and number density of the NWs as well as the 3D islands are listed in Table 1. It can be seen that the growth at the high deposition rate of 0.5 ML/min (Figure 4a) produced a large number of short NWs and small 3D islands. The number ratio of NWs to 3D islands is 1:2.3. The average length of the NWs and the average size of the 3D islands are about 126 nm and approximately 17 nm, respectively. At the high deposition rate, the Mn atoms have a short mean free path on the Si(110) surface and easily bind together or bind with the Si atoms to form the critical nuclei, leading to a high nucleation density. With decreasing Mn deposition rate, the number density of the NWs and 3D islands decreases significantly due to the low nucleation density. However, the average length of the NWs and the size of the 3D islands increase greatly. For example, at the low deposition rate of 0.02 ML/min (Figure 4d), the average length of the NWs and the size of the 3D islands are about 519 and 46 nm, respectively. Meanwhile, the number ratio of NWs to 3D islands is also increased to 1:1.3, indicating that a low deposition rate can restrain the nucleation of 3D islands and favor the formation of NWs. Compared to the high deposition rate, the increase in NW length and island size at the low deposition rate can be attributed to the longer growth time because the amount of deposited Mn is the same (1 ML).

**Table 1:** Average dimensions and number density of the NWs and 3D islands grown at different deposition rates

**Deposition rate (ML/min)**	**Length of NWs (nm)**	**Width of NWs (nm)**	**Height of NWs (nm)**	**Density of NWs (number/μm**^**2**^**)**	**Size of 3D islands (nm)**	**Height of 3D islands (nm)**	**Density of 3D islands (number/μm**^**2**^**)**
0.5	126.3	13.3	2.2	42	17.0	4.1	98
0.2	208.9	14.3	2.4	26	19.9	4.9	56
0.05	347.9	16.1	3.0	15	29.8	6.9	20
0.02	519.0	16.9	5.0	9	46.4	8.9	12

The growth temperature and Mn coverage for each deposition were kept at 550°C and 1 ML, respectively.

**Figure 5:**
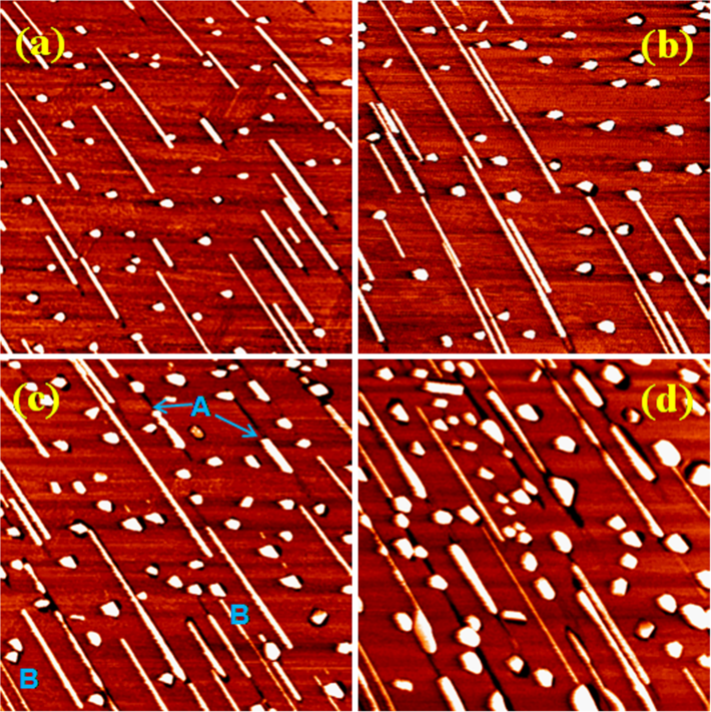
**The influence of deposition time on the growth of NWs.** Series of STM images (1,000 × 1,000 nm^2^) of the manganese silicide NWs and islands grown on the Si(110) surfaces at different durations. (**a**) 5, (**b**) 10, (**c**) 25, and (**d**) 50 min. The deposition rate and growth temperature were kept at approximately 0.2 ML min^−1^ and 550°C, respectively.

Figure 5 is a series of STM images showing the influence of deposition time (i.e., Mn coverage) on the growth of NWs, with the temperature and deposition rate kept at 550°C and 0.2 ML/min, respectively. The statistical results of the dimensions and number density of the NWs as well as the 3D islands are listed in Table 2. It can be seen that in the short-duration range (e.g., 5 and 10 min), the NWs formed on the surface are almost uniform in width and height, and the 3D islands are almost uniform in size, as shown by Figure 5a,b. The average length of the NWs and the average size of the islands are increased with the deposition time, as shown by Table 2. However, when the deposition time is increased to 25 min (Figure 5c), the NWs on the surface are no longer uniform in width and height. They exhibit two kinds of morphological changes. One is that some NWs begin to break and the fragments shrink into wider and higher elongated islands or 3D islands, leaving a narrow trough on the surface, as indicated by the label ‘A’. The other is that some NWs begin to dissolve and become thinner, with atoms diffusing to the nearby large islands, as indicated by the label ‘B’. This phenomenon is more obvious when the deposition time is increased to 50 min, as shown by Figure 5d. In addition, at the deposition time of 50 min, the 3D islands also become uneven in size. Figure 5 shows that with the continuous increase of deposition time, there is a trend for the NWs to evolve into large 3D islands, indicating that the NWs are a metastable silicide phase.

**Table 2:** Average dimensions and number density of the NWs and 3D islands grown at different deposition times

**Deposition time (min)**	**Length of NWs (nm)**	**Width of NWs (nm)**	**Height of NWs (nm)**	**Density of NWs (number/μm**^**2**^**)**	**Size of 3D islands (nm)**	**Height of 3D islands (nm)**	**Density of 3D islands (number/μm**^**2**^**)**
5	176.3	18.9	2.9	31	18.0	5.2	49
10	271.5	17.2	3.5	21	24.7	7.2	46
25	281.2	16.9	4.2	25	27.0	7.3	65
50	261.4	16.5	5.1	20	35.9	10.3	70

The growth temperature and deposition rate for each deposition were kept at 550°C and 0.2 ML/min, respectively.

As suggested in our previous studies, the formation mechanism of the Mn silicide NWs can be attributed to the anisotropic lattice mismatch between the Mn silicide and the Si(110) substrate [20,21]. In the width direction of NWs (i.e., Si[001] direction), the lattice mismatch has a relatively large value, and the adatoms are not easily attached to the two long edges of the NWs because of the high strain energy, leading to the limited growth along this direction. However, with extension of deposition time, more Mn atoms are supplied, and this will introduce dislocations in the NWs [9,27,28], resulting in the fragmentation of NWs and, finally, the reduction in their lengths. Meanwhile, the dislocations can relax the high strain along the width direction of NWs and thus make the adatoms attach to the wire edges more easily, leading to the increase in the wire width and height. The ‘A’-type change of the NWs shown in Figure 5c,d can be considered as a result induced by the dislocations. On the other hand, the appearance of ‘B’-type change of the NWs at a deposition time of 25 min (Figure 5c) indicates that the growth of NWs at this stage undergoes Ostwald ripening. Compared to large 3D islands, NWs have a large surface-to-volume ratio and thus a high chemical potential. According to the Gibbs-Thomson principle, the atoms would dissolve from thin NWs, diffuse over the surface, and finally attach to the large 3D islands, making the 3D islands become larger and the NWs become thinner until they disappear.

### Chemical composition of the NWs

The formation of Mn silicides on a Si substrate can be considered as a diffusion-determined chemical reaction between Mn and Si atoms [29]. The Si atoms that take part in the silicide reaction mainly come from the surface step edges or surface defects because the Si atoms at these places have less Si-Si bonds. In the above paragraphs, we mentioned that it is relatively easy to grow NWs with a large aspect ratio at a high temperature or a low Mn deposition rate. This fact indicates that the supply of sufficient free Si atoms per unit time plays an important role in the formation of NWs because more Si atoms can be detached from the substrate step edges at a high temperature, and the Mn atoms can encounter more Si atoms in the unit time at a low deposition rate. On the contrary, at a relatively low growth temperature or a high deposition rate, the supply of free Si atoms in the unit time is not sufficient and the formation of more 3D islands (Mn silicides rich in manganese) is the result. The Mn-Si binary alloy phase diagram shows that MnSi_~1.7_ is the only Si-rich silicide phase, and this phase is favored for high concentrations (≥50 at.%) of Si mixed with Mn at temperatures between approximately 400°C and 1,144°C [30]. Therefore, the Si-rich environment for the NW formation implies that the NWs are likely to be MnSi_~1.7_.

**Figure 6:**
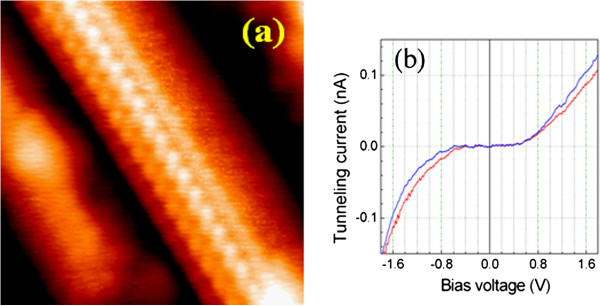
**Atomically resolved STM image of the manganese silicide NW and its tunneling current-voltage properties.** (**a**) Atomically resolved STM image (10 × 10 nm^2^) of an ultrafine manganese silicide NW grown on the Si(110) surface and **(b)** the scanning tunneling spectra measured on top of the NW showing semiconducting characteristics with a bandgap of approximately 0.8 eV. The red and blue curves were obtained on two different positions on the NW.

**Figure 7:**
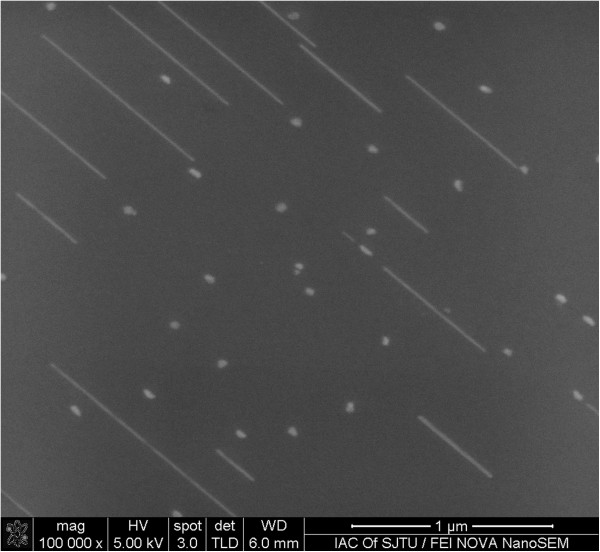
***Ex situ *** **BE-SEM image of the manganese silicide NWs and 3D islands grown on Si(110) surface.**

Figure 6a shows a high-resolution STM image of an ultrafine silicide NW grown on the Si(110) surface. A well-ordered atomic arrangement indicates that the silicide NW is single crystal. The atomic arrangement and the period of top atomic row in the wire direction, which is measured to be approximately 7.66 Å, are almost identical to those of the MnSi_~1.7_ NWs formed on a Si(111) surface [22]. The tunneling current-voltage (*I-V*) curves measured on top of the NW exhibit a semiconducting character with a bandgap of approximately 0.8 eV (Figure 6b), which is also consistent with that of the MnSi_~1.7_ NWs formed on the Si(111) surface [21]. Therefore, we deduce that the NWs formed on the Si(110) surface have the same composition as those formed on the Si(111) surface, i.e., the NWs are composed of MnSi_~1.7_. In order to further confirm this, we employed a BE-SEM to examine the chemical composition of the NWs formed on the Si(110) surface. The BE-SEM image provides an intensity map of the BE yield from the specimen. The BE yield increases with the atomic number of the elements encountered by the incident electron beam, i.e., compared to light elements, heavy elements yield more BEs. Therefore, the BE-SEM image reflects the distribution of chemical composition of the specimen. Figure 7 shows a BE-SEM image of the MnSi_~1.7_ NWs and the islands grown on the Si(110) surface. It can be seen that the NWs and 3D islands have sharply different contrast. The 3D islands are much brighter than the NWs, while the NWs are just a little brighter than the Si(110) substrate. This result indicates that the average atomic weight of the 3D islands is much greater than that of the NWs, while the average atomic weight of the NWs is slightly larger than that of the Si substrate. Therefore, the 3D islands and NWs have different chemical compositions. The 3D islands correspond to the Mn-rich silicide such as Mn_5_Si_3_, and the NWs correspond to the Si-rich phase MnSi_~1.7_. This conclusion is consistent with that reported for the Mn silicides formed on the Si(111) surface [20,21].

## Conclusions

In summary, the influence of growth conditions such as growth temperature, deposition rate, and deposition time on the formation of MnSi_~1.7_ NWs on a Si(110) surface has been investigated by STM. High growth temperature and low Mn deposition rate are found to be favorable for the formation of NWs with a large aspect ratio, indicating that the supply of free Si atoms per unit time plays a crucial role in the growth of the NWs. The NWs orient solely with the long axis along the Si $\left[1\bar{1}0\right]$ direction. The *I-V* curves measured on top of the NWs, and the BE-SEM image reveal that the NWs consist of MnSi_~1.7_. The growth of the parallel MnSi_~1.7_ NWs on the Si substrate provides an opportunity for the study of electronic properties of NWs and the fabrication of nanoelectronic devices with novel functions.

## Competing interests

The authors declare that they have no competing interests.

## Authors’ contributions

ZQZ designed the project of experiments and drafted the manuscript. WCL and XYL carried out the growth of MnSi_~1.7_ nanowires and STM measurements. GMS performed the SEM observations. All authors read and approved the final manuscript.
